# Comparison between Pressurized Liquid Extraction and Conventional Soxhlet Extraction for Rosemary Antioxidants, Yield, Composition, and Environmental Footprint

**DOI:** 10.3390/foods9050584

**Published:** 2020-05-05

**Authors:** Mathilde Hirondart, Natacha Rombaut, Anne Sylvie Fabiano-Tixier, Antoine Bily, Farid Chemat

**Affiliations:** 1 Avignon University, INRAE, UMR408, GREEN Team Extraction, F-84000 Avignon, France; mathilde.hirondart@sigma-clermont.fr (M.H.); rombaut.natacha@gmail.com (N.R.); anne-sylvie.fabiano@univ-avignon.fr (A.S.F.-T.); 2 ORTESA, LabCom Naturex-Avignon University, F-84000 Avignon, France; antoine.bily@givaudan.com; 3 Naturex-Givaudan, 250 rue Pierre Bayle, BP 81218, CEDEX 9, F-84911 Avignon, France

**Keywords:** Pressurized liquid extraction, soxhlet, solvent extraction, green analytical chemistry, Rosemary

## Abstract

Nowadays, “green analytical chemistry” challenges are to develop techniques which reduce the environmental impact not only in term of analysis but also in the sample preparation step. Within this objective, pressurized liquid extraction (PLE) was investigated to determine the initial composition of key antioxidants contained in rosemary leaves: Rosmarinic acid (RA), carnosic acid (CA), and carnosol (CO). An experimental design was applied to identify an optimized PLE set of extraction parameters: A temperature of 183 °C, a pressure of 130 bar, and an extraction duration of 3 min enabled recovering rosemary antioxidants. PLE was further compared to conventional Soxhlet extraction (CSE) in term of global processing time, energy used, solvent recovery, raw material used, accuracy, reproducibility, and robustness to extract quantitatively RA, CA, and CO from rosemary leaves. A statistical comparison of the two extraction procedure (PLE and CSE) was achieved and showed no significant difference between the two procedures in terms of RA, CA, and CO extraction. To complete the study showing that the use of PLE is an advantageous alternative to CSE, the eco-footprint of the PLE process was evaluated. Results demonstrate that it is a rapid, clean, and environmentally friendly extraction technique.

## 1. Introduction

In the field of raw material extraction, the first challenge consists of determining the potential of the plant matrix that means what can be extracted and valorized. The chemical composition of the plant material may highly vary depending on the local environmental conditions, development stages, plant part, harvesting season, the technique used for drying, and the storage condition. Therefore, for each batch of plant material used for industrial extraction, an analysis has to be performed to determine the amount of available extractives. 

In general, an analytical procedure for antioxidants from plants or spices comprises two steps: Extraction (Soxhlet, maceration, percolation) followed by analysis (spectrophotometry, high performance liquid chromatography coupled or not to mass spectrometry (HPLC-MS), gas chromatography coupled or not to mass spectrometry (GC–MS)). Whereas the last step is finished after only 15 to 30 min, extraction takes at least several hours. Conventional Soxhlet extraction (CSE) is the most used method for solid-liquid extraction in natural product chemistry and is a reference procedure for the extraction of fat and oil according to International Organization for Standardization (ISO standards) [1,2,3]. It has several disadvantages such as long operation time requiring a minimum of hours or days, large solvent volumes involved, time and energy consuming for the concentration step by evaporation to recover the final extract, and inadequacy for thermolabile analytes.

Pressurized liquid extraction (PLE) has been intensively studied as an efficient extraction technique to substitute CSE [2,4]. It is based on the ability to perform rapid (less than 30 min) and clean extraction at high pressure and temperature. Various parameters of extraction can be modified to improve extraction performance (solvent, pressure, temperature, time of extraction, etc.) [5,6,7,8,9]. High temperature and pressure increase analytes’ solubility and solvent diffusion rate, while solvent viscosity and surface tension decrease, resulting in a drained matrix after extraction [10]. With PLE, extractions can be programmed and automatically run, which is convenient for quality control.

**Figure 1:**
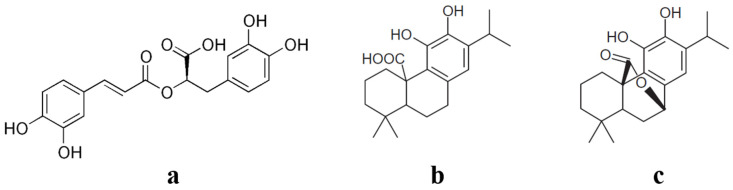
Structures of rosmarinic acid (**a**), carnosic acid, (**b**) and carnosol (**c**).

In this study we focused on rosemary (*Rosmarinus Officinalis* L.), which is mostly studied and used in the food industry due to its richness in antioxidants’ compounds [11,12], particularly rosmarinic acid (RA), carnosic acid (CA), and carnosol (CO) (Figure 1).

These compounds are extracted at industrial scale and are dedicated to food applications since the antioxidant extract of rosemary has been authorized in 2010 by the European Union as food additive E392 (directive No. 2010/69/EU). Throughout literature, extraction of chemical compounds from rosemary leaves has been investigated using PLE [13,14,15,16]. These studies were mainly focused on maximization of antioxidant activity of rosemary extracts and no complete parametric study of extraction of monitored compounds by PLE has been performed. Additionally, evaluation of the green aspects of PLE is not found in literature.

A major problem in the field of extraction remains the characterization of the raw material studied. The objective of our study was to propose a new method of raw material characterization by optimizing the extraction process in order to be sure to have exhausted the studied raw material. Numerous studies have already been carried out on the extraction of rosemary with innovative technologies such as supercritical fluid extraction (SFE) and pressurized liquid extraction (PLE) coupled with a new quantitative Ultra Performance Liquid Chromatography coupled to Tandem Mass Spectrometry (UPLC-MS/MS) method [13,14]. The difference with the work cited above is that we wanted to propose a green method that could replace Soxhlet in order to optimize the characterization of the raw material studied in the analytical laboratory. The procedure used minimizes the use of organic solvents, which makes it attractive in the analytical field.

In the present work, PLE was studied as a green alternative to Soxhlet extraction of antioxidants from rosemary leaves to extract qualitatively and quantitatively RA, CA, and CO. PLE was optimized via a response surface methodology and a desirability function, which simultaneously maximized extraction, was used. We ran statistical tests in order to check the reliability and the reproducibility of this new procedure. Finally, the eco-footprint of the PLE process was evaluated to demonstrate that it is a rapid, environmentally friendly, and clean extraction technique.

## 2. Materials and Methods 

### 2.1. Plant Material and Chemicals

Rosemary leaves (*Rosmarinus officinalis* L.) were provided by the company Naturex (Avignon France), and rosemary leaves were collected in Morocco in 2015. Initial moisture was 8.2 ± 0.2%. Leaves were ground before extraction using a grinder (MF 10 basic, IKA, Staufen, Germany) with a 0.5-mm sieve. Granulometry of the rosemary powder was 610 ± 22 µm.

For the extraction solvent, food grade ethanol 96° *v*/*v* (Cristalco, FranceAlcools, Paris, France) and demineralized water were used. For HPLC analysis, solvent used were all HPLC grade: Methanol, water, acetonitrile, and tetrahydrofuran. Phosphoric acid 85% ACS grade (according to American Chemical Society specifications) and trifluoroacetic acid 99% were purchased from Sigma-Aldrich, USA. Standards used were rosmarinic acid (Extrasynthese, Genay, France) and carnosic acid (Sigma-Aldrich, St. Louis, MO, USA). Nitrogen used had a purity of 99.999% (Alphagaz 1 Smartop, Air Liquid, Paris, France).

### 2.2. Extraction Procedures

**Figure 2:**
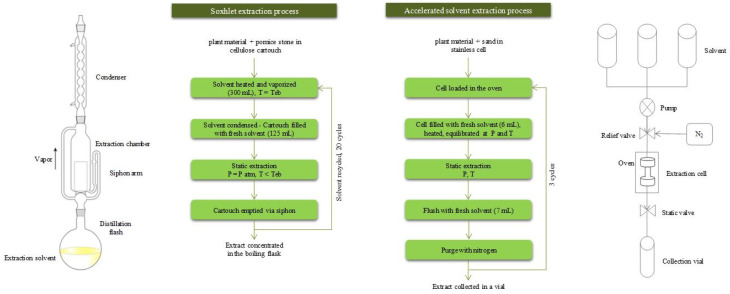
Comparison of Soxhlet and Accelerated Solvent Extraction (ASE) processes.

In this study, a procedure of PLE was developed and optimized for analytical determination of RA and CA contents in rosemary leaves. PLE performance was compared to the reference method of Soxhlet extraction. Those processes are illustrated in Figure 2.

#### 2.2.1. Reference Procedure: Conventional Soxhlet Extraction (CSE)

For CSE, 10 g of ground rosemary leaves and 5 g of pumice stone were mixed in a 34 × 130 mm cellulose thimble (plugged with cotton in order to avoid transfer of sample particles in the distillation flask) and placed in Soxhlet apparatus with flask containing 300 mL of solvent. Extractions were performed using a solid to liquid ratio of 1 to 12 (g/mL). Extraction was performed during 8 h. After extraction, the extract was concentrated under vacuum (Laborota 4001, Heidolph, Germany) and conserved at 4 °C before analysis. All extractions were done at least in duplicate and the mean values were reported.

#### 2.2.2. Pressurized Liquid Extraction (PLE)

An accelerated solvent extractor ASE200 model was used (Dionex, Thermo Fisher Scientific, Waltham, MA, USA). This apparatus allows extraction of plant material at high pressure (up to 130 bar) and high temperature (up to 200 °C). Preliminary trials were made in order to determine the optimal parameters (loading of the cell, flushing volume, and percentage of dispersant), and will be discussed in the result section. Optimal loading was determined to be 3.1 g of ground rosemary leaves, and 7.3 g of Fontainebleau sand (VWR Chemicals, Radnor, PA, USA) were homogenized in an 11-mL stainless-steel cell. The cells were equipped with stainless steel frits on both sides, and a cellulose filter at the bottom to obtain a filtered extract. The extraction procedure cycle was done as follows: First, the cell was filled with extraction solvent via an HPLC pump, pressurized, and placed into the preheated oven. Depending on the set extraction temperature, the cell preheating duration was between 5 and 9 min, followed by a static period of extraction. Then, the cell was flushed with fresh solvent (60% of the extraction cell volume) and purged with a flow of nitrogen during 1 min. Several cycles of extraction can be performed to drain active compounds from the plant matrix. Extracts were collected into a glass vial and analyzed without a concentration step. The dry matter content of each extract was determined by drying 5 mL of extract at 130 °C during 3 h, to calculate the mass extraction yield. 

Preliminary trials were performed to evaluate the impact of some PLE parameters on extraction performance: Solvent, percentage of dispersant, and flushing volume. For these trials, the other extraction parameters were fixed according to literature [17]: Temperature (T) = 100 °C, Pressure (P) = 80 bar, static time of extraction = 5 min, and 3 cycles of extraction.

### 2.3. Statistical Analysis

#### 2.3.1. Experimental Design 

To investigate the influence of PLE extraction parameters on the extraction of rosemary antioxidants, a response surface methodology was used. Three independent factors, namely the temperature (A), the pressure (B), and the extraction time (C), were studied to evaluate their impact on several responses: The mass yield (%) and the contents in RA, CA, and CO (mg/g). The independent variables, given in Table 1, were coded according to Equation (1):

(1) $$X_{i}=\frac{x_{i}-x_{i0}}{\Delta x_{i}}$$

where *X_i_* and *x_i_* are, respectively, the dimensionless and the actual values of the independent variable *i*, *x_i_*_0_ is the actual value of the independent variable *i* at the central point, and Δ*x_i_* is the step change of *x_i_* corresponding to a unit variation of the dimensionless value. For the three variables, the design yielded randomized experiments with eight (2^3^) factorial points, six axial points (−*α* and +*α* (in our case 1.68)) to form a central composite design, and six center points for replications and estimation of the experimental error and to prove the suitability of the model. Coded values of the independent variables are listed in Table 1.

(2) $$Y=\beta_{0}+\overset{2}{\underset{i=1}{\sum }}\beta_{i}X_{i}+\overset{2}{\underset{i=1}{\sum }}\beta_{ii}X_{i}^{2}+\underset{i}{\sum }\underset{j=i+1}{\sum }\beta_{ij}X_{i}X_{j}$$

The responses are related to the coded independent variables *X_i_* and *X_j_* according to the second order polynomial expressed in Equation (2) with *β*_0_ the interception coefficient, *β_i_* the linear terms, *β_ii_* the quadratic terms, and *β_ij_* the interaction terms. Fisher’s test for analysis of variance (ANOVA) performed on experimental data was used to assess the statistical significance of the proposed model. The experimental design was analyzed using the software Statgraphics (StatPoint Technologies, Inc., Warrenton, VA, USA) for Windows.

#### 2.3.2. Reproducibility and Statistical Comparison

The optimized PLE method compared to the CSE method was performed for the extraction of antioxidants from rosemary. It consisted of a series of eight successive experiments performed for each extraction procedure. Then the statistical study was performed in two steps: First, the Fisher–Snedecor’s test to compare the variability of the results and then the student test in order to compare the mean values obtained by the two different extraction procedures. Those two tests were performed with α = 0.05.

### 2.4. HPLC Analysis 

Analyses of RA, CA, and CO were performed by HPLC (Agilent 1100, Agilent Technologies, Santa Clara, CA, USA) equipped with a Diode Array Detector (DAD) detector. HPLC analyses were made according to previously reported procedures without further optimization and specific procedures for each compound are described below [18].

#### 2.4.1. Rosmarinic Acid Analysis

The column used was a C_18_ column (5 µm, 4.6 mm × 250 mm, Zorbax SB, Agilent Technologies, Santa Clara, CA, USA). The mobile phase was composed of 32% acetonitrile and 68% water with 0.1% trifluoroacetic acid (mL/mL) and the flow rate was set at 1 mL/min. The column oven temperature was 20 °C and the run time was 10 min. Five µL were injected. Rosmarinic acid was detected at a wavelength of 328 nm. For quantification of rosmarinic acid in the extract, a calibration curve was calculated by linear regression analysis for rosmarinic acid standard.

#### 2.4.2. Carnosic Acid and Carnosol Analysis

The column used was a C_18_ column (1.8 µm, 4.6 mm × 50 mm, Zorbax Eclipse XBD-C18, Agilent Technologies, France). The mobile phase was isocratic and composed of 0.5% H_3_PO_4_ (in water)/acetonitrile (35/65, mL/mL), and the flow rate was set at 1.5 mL/min. The column oven temperature was 25 °C and the run time was 15 min. Five µL were injected. Carnosic acid and carnosol were detected at a wavelength of 230 nm. For quantification of carnosic acid in the extract, a calibration curve was calculated by linear regression analysis for carnosic acid standard. Carnosol was expressed as carnosic acid.

### 2.5. Calculations

In order to assess the extraction performances of the evaluated processes, mass extraction yield, purity, and content in each compound of interest were calculated. Each mass included in equations below was expressed in dry weight.

(3) $$mass\;extraction\;yield\;\left(\%,\frac{g}{100g}\right)=\frac{weight\;of\;extract}{weight\;of\;rosemary\;leaves\;}\times \;100$$

(4) $$purity\;\left(\%,\frac{g}{100g}\right)=\frac{weight\;of\;RA,\;CA\;or\;CO}{weight\;of\;extract\;}\times \;100$$

(5) $$content\;in\;RA,\;CA\;and\;CO\;\left(mg/g\;rosemary\right)=\frac{purity\;\times \;weight\;of\;extract}{weight\;of\;rosemary\;leaves}$$

## 3. Results and Discussion

### 3.1. Pressurized Liquid Extraction (PLE): Preliminary Study

#### 3.1.1. Solvent Evaluation of Ethanol/Water Ratio on Extraction Efficiency

To determine the solvent that maximized the extraction of both RA and CA, PLE was performed with various percentages of ethanol in water: 0, 20, 40, 60, 80, and 100% (g/g). Hydro-alcoholic solutions as solvent offer many advantages. Indeed, they can solubilize both hydrophilic and lipophilic active compounds. To test different ethanolic solvent proportions, the flushing volume was fixed at 60% mL/mL of the extraction cell. The extraction cell was filled with 30% g/g of ground rosemary leaves and 70% g/g of Fontainebleau sand. 

**Figure 3:**
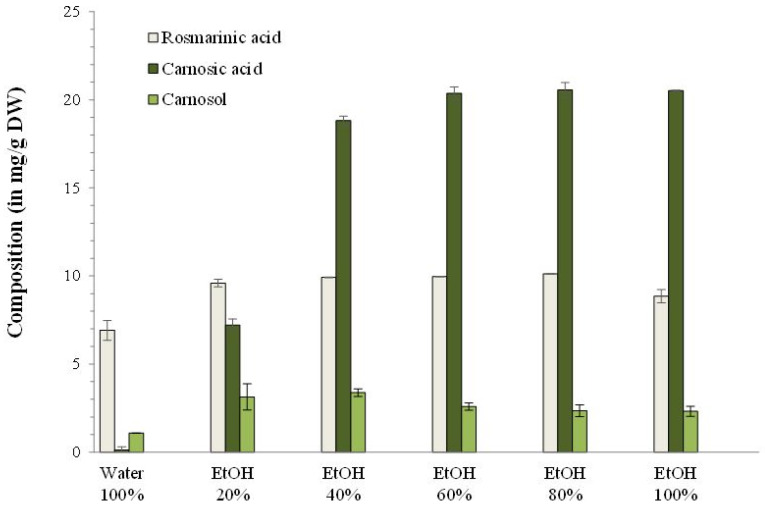
Influence of different ratios of ethanol/water as extraction solvent on the antioxidants composition of Pressurized Liquid Extraction (PLE) extracts of rosemary.

The influence of ethanol proportion in the extraction solvent on extract composition is reported in Figure 3. 

At low ethanol percentage (0 and 20%), RA was extracted but no CA, while from 40% ethanol, CA was extracted as well. These results showed that the solvent maximizing both RA and CA extraction was 80% ethanol, with 10.13 ± 0.02 mg RA/g rosemary and 20.6 ± 0.4 mg CA/g rosemary extracted. Maximal extraction and solubilization of both compounds was possible with 80% ethanol thanks to its intermediate polarity, despite the different chemical structures of RA and CA. Indeed, RA is a caffeic acid ester [19], rather hydrophilic, so preferentially extracted and solubilized in solvents that are relatively polar, as ethanol [20]. CA is a phenolic diterpene [21] and is relatively lipophilic, but still soluble in intermediate polarity solvents such as acetone or ethanol [22,23].

#### 3.1.2. Dispersant

**Figure 4:**
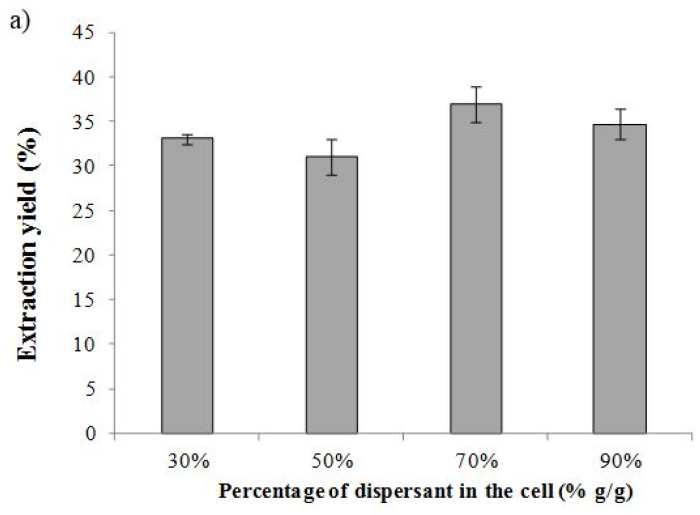

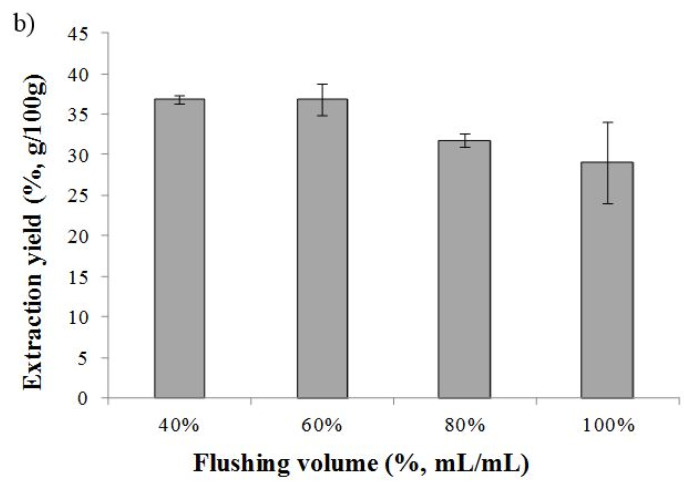
Influence of dispersant proportion in the extraction cell (**a**) and flushing volume (**b**) on the mass extraction yield of PLE of rosemary leaves. The bars with range mean standard deviation between three experiments.

Fontainebleau sand was used as a dispersant in order to favor a uniform distribution of sample and maximize the extraction yield. It was mixed with ground rosemary leaves in different proportions to quantify the impact of dispersant on extraction yield. Trials were carried out with 30, 50, 70, and 90% g/g of dispersant. This parameter is usually fixed or not specified in literature, suggesting that it is not impacting the extraction performances. In this study, we measured its impact only on mass yield to verify this hypothesis. The flushing volume was fixed at 60% mL/mL of the extraction cell, and 80% ethanol was used as extraction solvent. It can be seen in Figure 4 that the proportion of dispersant in the cell had a small impact on extraction mass yield, which varied between 31 ± 2% and 37 ± 2%. The 70% dispersant must be selected to maximize the mass yield (37 ± 2%).

#### 3.1.3. Flushing Volume

The flushing volume is the amount of fresh solvent injected during PLE after static extraction (Figure 2). It is measured as a percentage mL/mL of the extraction cell volume (11 mL). Extractions were performed with 40, 60, 80, and 100% mL/mL (Figure 4). 

As for the proportion of dispersant, the flushing volume is usually fixed in literature [14,15]. In this study we measured its impact on extraction mass yield. A flushing volume of 60% mL/mL maximized the extraction mass yield (36.9 ± 2.0%), while higher flushing volume decreased it. A 60% flushing volume was commonly applied throughout literature, which confirms the results obtained [14].

### 3.2. PLE Extraction: Experimental Design and Statistical Analysis

Three variables that could impact extraction efficiency of antioxidants from rosemary by PLE were studied in a central composite design, namely, temperature (A), pressure (B), and extraction time (C). The choice of the A and B variation domain was selected considering the limits of the ASE equipment. A range from 40 to 190 °C was chosen for the temperature (A), and a range from 40 to 130 bar for the pressure (B). This wide temperature range was chosen to thoroughly evaluate temperature impact on extraction. Within this range, thermal degradation of compounds could also be assessed. The total extraction duration depends on the duration of equilibration of the cell, which varies from 5 to 9 min according to the temperature of extraction. However, as the temperature is a factor impacting the extraction, we chose to consider the duration of static extraction as an independent variable (C). As we performed three cycles of PLE for each extraction cell (Figure 2), the extraction time (C) was the sum of the three static extraction periods. A range from 3 = 3 × 1 min to 33 = 3 × 11 min of static extraction was selected. The controlled variables were studied in a multivariate study with 20 experiments, as shown in Table 1. 

Responses varied greatly as a function of the combination of parameter settings. Mass extraction yield ranged from 26.3 to 49.6%, RA content ranged from 9.93 mg/g rosemary to 12.24 mg/g rosemary, and CA content ranged from 19.90 mg/g rosemary to 22.12 mg/g rosemary (Table 1). Experimentally, the formation of Maillard reaction product at temperature of 150 °C or above occurred as evidenced by the brown color of the extract and the burnt smell. The presence of these toxic compounds in extracts must be avoided, so extractions carried out at temperature in the range of 150–200 °C are usually not recommended [15]. However, in this study extraction was investigated for analytical purpose and sensory characteristics of the extracts was not considered. Moreover, the degradation of targeted compounds did not occur because the RA and CA content in extracts were not lower at 190 °C than at 115 °C (Table 1). As shown in Table 1, there was no significant difference in CO content, which indicates that there was no degradation of CA into CO due to oxidation, as it is suggested in published studies [18,22].

By considering a confidence level of 95%, the linear effects of the temperature (A) as well as all quadratic effects (A^2^, B^2^, and C^2^) were significant (Table 2) with *p*-value below 0.05. There were also interactions between variables A and B in significant scale, with a *p*-value lower than 0.05 (Table 2). Empirical relationships allowed linking responses studied and key variables involved in the model. From ANOVA, the coefficient of determination *R*^2^ was determined to be higher than 80% for the three considered responses.

**Figure 5:**
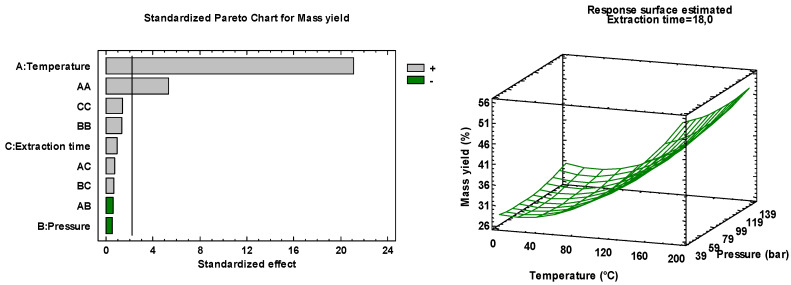

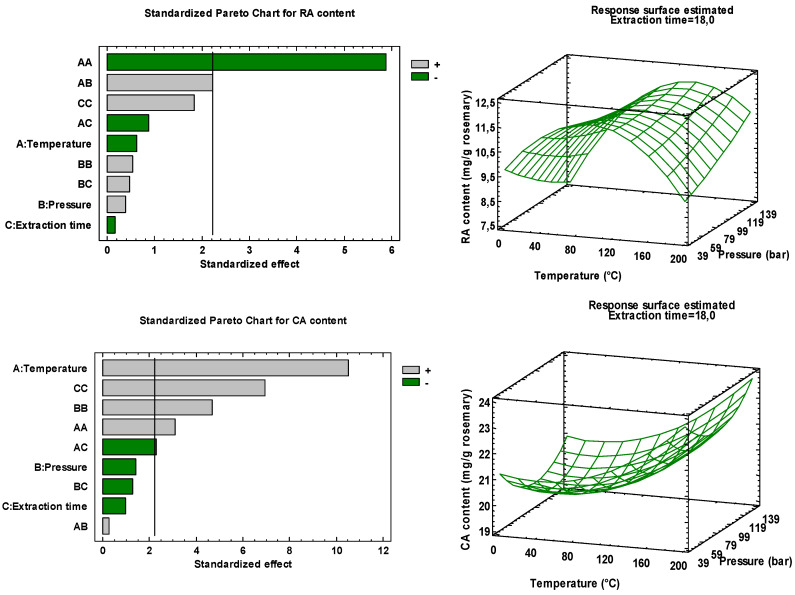
Standardized Pareto charts and response surfaces estimated for the optimization of PLE parameters.

Three-dimensional surface responses of a multiple nonlinear regression model (Figure 5) illustrate the linear and quadratic effects together with the interaction effects on the responses given in Table 1. Figure 5 highlights the behavior of the three responses as a function of two variables: Temperature (A) and pressure (B). In each plot, the extraction time (C) was fixed at the central value (“0”). The most influential effect was the linear terms of temperature (A) as can be seen in Table 2, with low *p*-values: 0, 0.5871, 0 for mass yield, RA content, and CA content, respectively. 

As expected, the model confirmed that mass extraction yield increased with temperature (B). Influence of quadratic terms given in Table 2 is illustrated in Figure 5 by observation of the surface curvatures of the plots. Optimal settings for the maximization of each response are presented in Table 2. An optimization of the desirability was carried out to obtain optimal factors’ settings for the multi-responses’ maximization. The settings which simultaneously maximized mass yield, RA content, and CA content were: Temperature A = 183 °C, pressure B = 130 bar, extraction time C = 3 × 1 min. 

### 3.3. Statistical Comparison of CSE and PLE

In order to assess the reliability of the PLE extraction technique to replace CSE for raw material active content determination, statistical analysis was performed on repeated trials. For this purpose, 16 experiments were run, 8 with the conventional Soxhlet technique and 8 with the optimized PLE extraction (Table 3). The RA, CA, and CO contents (mg/g rosemary) were analyzed and reported. 

The mean value of RA content obtained with PLE was similar to the value obtained using CSE (10 ± 1 and 9.9 ± 0.5 mg/g rosemary, respectively) and the mean value of CA content obtained with PLE was higher than the value obtained using CSE (21 ± 1 and 17.7 ± 0.9 mg/g rosemary, respectively). The relative standard deviations were 10% for PLE and CSE, which means that the results were little dispersed around the mean value. This was confirmed using the Fisher–Snedecor’s test, which gave no significant difference in the dispersion of the results for the two different processes (α = 0.05) for both RA and CA content. The student test was then applied in order to check if there was any significant difference between the mean values of RA and CA content obtained by the two processes. The tabular value obtained by the student test table for α = 0.05 was 2.10 and the calculated value was 0.35 for RA content and 1.83 × 10^−6^ for CA content, which meant that there was no significant difference between the mean values from a statistical point of view. The statistical tests validated the PLE technique as a good alternative to the conventional one for the determination of active compounds’ content in rosemary.

Extraction performance of CSE and PLE are compared in Table 3 in terms of mass extraction yield and active contents. Mass yield of optimized PLE extraction (47.6 ± 0.5%) was higher than mass yield obtained with CSE (26 ± 1%). High pressure and high temperature generated during PLE enabled extracting more compounds from the plant material [16]. Extraction of active compounds such as RA and CA was improved with PLE (Table 3). The use of drastic extraction conditions during PLE did not lead to the degradation of compounds, even the most thermosensitive such as CA. This absence of degradation could be explained by the absence of oxygen during PLE due to nitrogen flushing and a short contacting duration between the solvent and the matrix (around 45 min against 8 h with CSE). A higher CO content was obtained with CSE than with PLE, respectively 4.4 ± 0.8 mg/g rosemary and 1.9 ± 0.3 mg/g rosemary, suggesting the degradation of CA into CO during CSE (Table 3). Due to higher extraction yields achieved with PLE, purity of the active compounds in extracts was lower, this extraction technique was less selective regarding these compounds. However, the goal of this analytical technique was not to reach high purities of RA and CA in extracts, but to drain completely the plant material. High purity in RA and CA in the final extract was not considered as a response to maximize. PLE with optimized conditions seems to be a good technique to quantitatively extract RA, CA, and CO from ground rosemary leaves, with better performance than CSE in term of active content yields.

### 3.4. Eco-Footprint: CSE vs. PLE Processes

The two extraction techniques were evaluated according to the six principles of green extraction developed by Chemat et al. [24]. The six parameters considered were calculated as follows:

- Raw material (Principle 1): Mass of plant material required for an analysis (in g).
- Solvent (Principle 2): Mass of solvent required for an analysis (in g).
- Energy (Principle 3): Energy consumption for the analysis considering extraction and evaporation steps based on the energy transfer equation [25,26,27] (in kWh).
- By-products (Principle 4): Amount of waste generated by an analysis (solvent and plant material) (in g).
- Process (Principle 5): Time of an analysis including steps of preparation, extraction, evaporation, and cleaning (in h).
- Product recovery (Principle 6): (Mass of final product recovered) / (mass of available product in the plant material) (in %).

**Figure 6:**
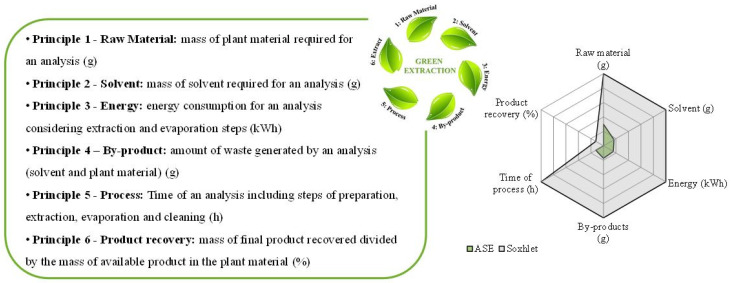
Eco-footprint of PLE vs. CSE processes.

In Figure 6, it is important to notice that for each principle, a value close to the center is a positive result whereas a value far from the center corresponds to a negative result. Thus, for “Product recovery”, the center corresponds to a maximum of actives extracted.

Compared to CSE, PLE enabled reducing extraction time by a factor 8, from 9 h 40 min to 1 h 10 min. As well, PLE required less solvent, around 50 mL against 300 mL for CSE. Waste of solvent is a real problem in analytical laboratories because usually solvents are not recycled. It is all the more important to minimize the amount of solvent required for an analysis. Thus, during PLE less waste is produced by an analysis in terms of solvent and spent residue. In PLE extraction, less raw material was needed (3 g against 10 g for CSE). 

More than the economical aspect, it can be very practical in a sourcing demarche where, regularly, only few quantities of raw material are available. Energy consumption was lower for PLE extraction, because even if the extraction temperature was higher (183 °C instead of 78 °C for CSE), less solvent had to be heated (50 mL against 300 mL), and there were only 3 heating cycles during PLE against 20 cycles during CSE. Another positive aspect of PLE compared to CSE was the percentage of product recovery (100% for PLE and 87.7% for CSE). Higher pressure and temperature during PLE allowed extracting more actives, and their degradation was avoided thanks to the absence of oxygen in the system and the short time of contact between the matrix and the solvent. Finally, the reduced cost of extraction was advantageous for the PLE method in terms of time, amount of raw material and solvent, product recovery, and waste generated. The eco-footprint of PLE was 33 times lower than CSE, with 2.96 area units for PLE against 100 area units for CSE, represented in Figure 6. The implementation of this technique in industrial quality control laboratories could be advantageous compared to CSE in terms of capital expenses and economic savings. 

## 4. Conclusions

Optimization of PLE was carried out using a central composite design methodology. Maximization of extraction was obtained combining three PLE parameters: Temperature (183 °C), pressure (130 bar), and static duration of extraction (3 min). Given the high temperatures tested, carnosol was monitored to follow degradation of carnosic acid and no increase of carnosol was evidenced. To evaluate if PLE could replace CSE, a statistical comparison of extraction performances of the two processes was performed. Ultimately, the eco-footprint of PLE and CSE were determined considering consumption of raw material, solvent, energy, and time. PLE proved to be a rapid, clean, and environmentally friendly technique for determination of active content in plant matrices. 

## Figures and Tables

**Table 1:** Central composite design (CCD) matrix with experimental responses obtained (mass extraction yield and leaf content in rosmarinic acid, carnosic acid, and carnosol).

Variables						Responses			
Temperature		Pressure		Extraction Time		Mass Yield	RA Content	CA Content	CO Content
Actual Value (°C)	Coded Value	Actual Value (bar)	Coded Value	Actual Value (min)	Coded Value	%	mg/g	mg/g	mg/g
160	+1	111.8	+1	27	+1	44.0	11.56	21.76	2.01
160	+1	111.8	+1	9	−1	41.9	11.57	22.54	2.10
160	+1	58.2	-1	27	+1	44.9	11.18	22.32	2.07
160	+1	58.2	−1	9	−1	44.0	11.49	22.62	2.17
70	−1	111.8	+1	27	+1	31.3	11.13	20.55	2.22
70	−1	111.8	+1	9	−1	30.5	10.83	20.49	2.40
70	−1	58.2	−1	27	+1	31.3	11.84	21.20	2.16
70	−1	58.2	−1	9	−1	31.5	11.67	20.68	2.12
115	0	85	0	18	0	34.3	11.29	20.98	2.17
115	0	85	0	18	0	34.5	11.70	20.12	2.17
115	0	85	0	18	0	34.3	11.72	20.63	2.05
115	0	85	0	18	0	34.8	11.71	20.41	2.11
115	0	85	0	18	0	34.3	11.68	20.39	2.18
115	0	85	0	18	0	34.1	12.01	20.34	2.09
39.3	−α	85	0	18	0	26.3	10.56	19.90	2.29
190.7	+α	85	0	18	0	49.6	9.93	22.12	2.22
115	0	40	−α	18	0	34.0	11.32	21.28	2.30
115	0	130	+α	18	0	35.1	12.24	21.35	1.98
115	0	85	0	3	−α	34.5	12.19	21.88	2.07
115	0	85	0	33	+α	34.7	11.99	21.63	2.10

**Table 2:** Summary of the ANOVA for the central composite design.

Variables	Responses					
	Mass Yield		RA Content		CA Content	
	*F-Ratio*	*p-Value*	*F-Ratio*	*p-Value*	*F-Ratio*	*p-Value*
Temperature (A)	445.98	0	0.31	0.5871	106.25	0
Pressure (B)	0.26	0.6225	0.12	0.7389	2.12	0.1756
Extraction time (C)	0.86	0.3743	0.04	0.8487	0.89	0.3685
A^2^	28.41	0.0003	32.84	0.0002	9.79	0.0107
A × B	0.38	0.5508	5.17	0.0463	0.07	0.7911
A × C	0.55	0.4758	0.57	0.4661	4.74	0.0546
B^2^	1.81	0.208	0.19	0.6738	21.88	0.0009
B × C	0.46	0.5125	0.29	0.6002	1.18	0.3020
C^2^	1.97	0.1904	3.50	0.0907	49.02	0
Error R^2^ (%)	97.9518		82.1394		94.8633	
R^2^ adjusted for d.f (%)	96.1084		66.0649		90.2402	
Optimal conditions predicted	A = 190 °C B = 40 bar C = 33 min		A = 100 °C B = 40 bar C = 3 min		A = 190 °C B = 130 bar C = 4 min	

**Table 3:** Reproducibility of extraction and statistical comparison test between Pressurized Liquid Extraction (PLE) and Conventional Soxhlet Extraction (CSE).

Experiments		1	2	3	4	5	6	7	8	Mean (%)	SD (%)	RSD (%)	Variance S^2^	F_CAL_	F_TAB_	t_CAL_	t_TAB_
**Extraction yield (%)**	CSE	26.5	26.2	28.9	26.3	28.2	28.3	28.7	30.2	27.91	1.45	5.21	1.85	0.31	3.79	2.55 × 10^−14^	2.1
	PLE	47.8	44.7	46.7	46.2	46.5	45.5	45.3	46.7	46.20	0.97	2.11	0.83				
**RA content (mg/g)**	CSE	9.71	9.23	9.98	9.14	9.70	10.39	10.23	10.49	9.86	0.51	5.13	0.22	0.11		0.35	
	PLE	11.74	10.88	9.15	9.95	8.59	9.96	9.87	9.92	10.01	0.97	9.65	0.82				
**CA content (mg/g)**	CSE	18.18	17.08	17.38	17.56	17.61	16.16	18.67	18.78	17.68	0.86	4.88	0.65	0.69		1.83 × 10^−6^	
	PLE	22.46	20.18	22.38	20.62	21.26	19.99	20.25	21.88	21.13	1.01	4.78	0.89				
**CO content (mg/g)**	CSE	3.51	4.29	4.34	5.64	4.25	5.36	3.62	4.14	4.39	0.75	17.14	0.50	0.01		1.67 × 10^−7^	
	PLE	2.20	1.72	1.69	2.00	1.68	1.96	1.50	2.16	1.86	0.25	13.58	0.06				

SD, standard deviation; RSD, relative standard deviation; F_CAL,_ F value calculated using Fisher-Snedecor’s test; F_TAB_, F value tabulated for α = 0.05 and 7 of freedom; t_CAL_ = *t* value calculated using student’s test; t_TAB_ = *t* value tabulated for α = 0.05 and 14 degrees of freedom.
